# Supplementary material for: 4-[(E)-2-(1-Pyrenyl)Vinyl]Pyridine Complexes: How to Modulate the Toxicity of Heavy Metal Ions to Target Microbial Infections
Source: Molecules. 2024 Mar 31;29(7):1565. doi: 10.3390/molecules29071565 (PMC11013842; doi:10.3390/molecules29071565)
Supplement: Supplementary file 1 [file molecules-29-01565-s001.zip › molecules-2925759-supplementary.pdf]

molecules

Article

4-[(E)-2-(1-Pyrenyl)Vinyl]Pyridine Complexes: How to Modulate the Toxicity of Heavy Metal Ions to Target Microbial Infections

Justine V. Schwarte [1], Aurélien Crochet [1,2] and Katharina M. Fromm [1,2,3,*]

1 Department of Chemistry, University of Fribourg, Chemin du Musée 9, 1700 Fribourg, Switzerland; justine.schwarte@unifr.ch (J.V.S.); aurelien.crochet@unifr.ch (A.C.)
2 Fribourg Center for Nanomaterials, 1700 Fribourg, Switzerland
3 NCCR Bio-Inspired Materials, University of Fribourg, 1700 Fribourg, Switzerland
* Correspondence: katharina.fromm@unifr.ch

Abstract: Pyrene derivatives are regularly proposed for use in biochemistry as dyes due to their photochemical characteristics. Their antibacterial properties are, however, much less understood. New complexes based on 4-[(*E*)-2-(1-pyrenyl)vinyl]pyridine (PyPe) have been synthesized with metal ions that are known to possess antimicrobial properties, such as zinc (II), cadmium (II), and mercury (II). The metal ion salts, free ligand, combinations thereof, and the coordination compounds themselves were tested for their antibacterial properties through microdilution assays. We found that the ligand is able to modulate the antibacterial properties of transition metal ions, depending on the complex stability, the distance between the ligand and the metal ions, and the metal ions themselves. The coordination by the ligand weakened the antibacterial properties of heavy metal ions (Cd(II), Hg(II), Bi(III)), allowing the bacteria to survive higher concentrations thereof. Mixing the ligand and the metal ion salts without forming the complex beforehand enhanced the antibacterial properties of the cations. Being non-cytotoxic itself, the ligand therefore balances the biological consequences of heavy metal ions between toxicity and therapeutic weapons, depending on its use as a coordinating ligand or simple adjuvant.

Keywords: pyrene derivatives; antimicrobial compounds; metal ion toxicity; pyrene-complexes; co-administration; adjuvant; synergy

Citation: Schwarte, J.V.; Crochet, A.; Fromm, K.M. 4-[(E)-2-(1-Pyrenyl) Vinyl]Pyridine Complexes: How to Modulate the Toxicity of Heavy Metal Ions to Target Microbial Infections. *Molecules* **2024**, *29*, 1565. https://doi.org/10.3390/molecules29071565

Academic Editor: Mauro Ravera

Received: 5 March 2024
Revised: 23 March 2024
Accepted: 26 March 2024
Published: 31 March 2024

Copyright: © 2024 by the authors. Licensee MDPI, Basel, Switzerland. This article is an open access article distributed under the terms and conditions of the Creative Commons Attribution (CC BY) license (https://creativecommons.org/licenses/by/4.0/).

1. Introduction

The increase in bacterial resistance to more and more antibiotics is a worrying result of the globalization and excess use of these antibiotics [1,2]. It has become urgent to find new antibiotic families to circumvent bacterial recognition [3], and/or to impair bacterial resistance pathways. While traditional antibiotics target specific binding sites in bacteria, metal ions with antibacterial properties such as bismuth(III), silver(I), and gold(I) usually attack at several different places in a cell, and their coordination by ligands can help make targeting more specific [4–8].

There are nevertheless two issues concerning the use of metal ions as therapeutic weapons. The first concerns the development of resistance pathways to these ions by some bacteria, generally through (over-)expression of efflux pumps [9], transporters [10,11], or chaperones [10] (for a complete review, see [12]). Secondly, bacteria are not always the only susceptible organisms to metal ions, and some heavy metal ions are toxic to mammalian cells, e.g., cadmium(II), arsenic(III or V), and mercury(II) [13–17]. However, the association of these ions to organic compounds as ligands can sometimes attenuate the toxicity towards human/eukaryotic cells without affecting bacterial susceptibility. Some examples include merbromin and thimerosal, organomercuric compounds used, respectively, as topical antiseptic and preservative [18–20]; padeliporfin, which is based on palladium and targets cancerous cells [21]; stibogluconate, based on antimony and used

against Leishmaniasis [22]; and the arsenic-based tuberculosis treatment, arsinothricin [23].

These examples illustrate the renewed research interest in associating metal ions with antibiotic molecules for a synergic effect [23–26]. As the final compounds are of different sizes and possess different chemical and physical properties than the starting compounds, the individual components of the coordination compound can mutually benefit from a non-recognition of their respective resistance markers. Moreover, as they usually possess different antibacterial mechanisms, the bacteria are attacked in different ways.

Herein we studied how a pyrene-pyridine ligand (PyPe) can influence the minimum inhibitory concentration (MIC) of different heavy metal ions, although PyPe alone does not display any antibacterial effect on its own.

2. Results

2.1. Chemistry

2.1.1. Synthesis of the Ligand

To prepare the pyrene-pyridine ligand, pyridine-4-ylmethanide was prepared in situ by adding a solution of *n*-BuLi and diisopropylamine to a solution of 4-picoline. Then, 1-pyrenecarboxaldehyde was added to the resulting solution, forming the intermediate alcohol **1** [27]. Dehydration was then managed with a solution of $POCl_3$, followed by the basification of the mixture, resulting in the precipitation of pure PyPe (Figure 1) [27].

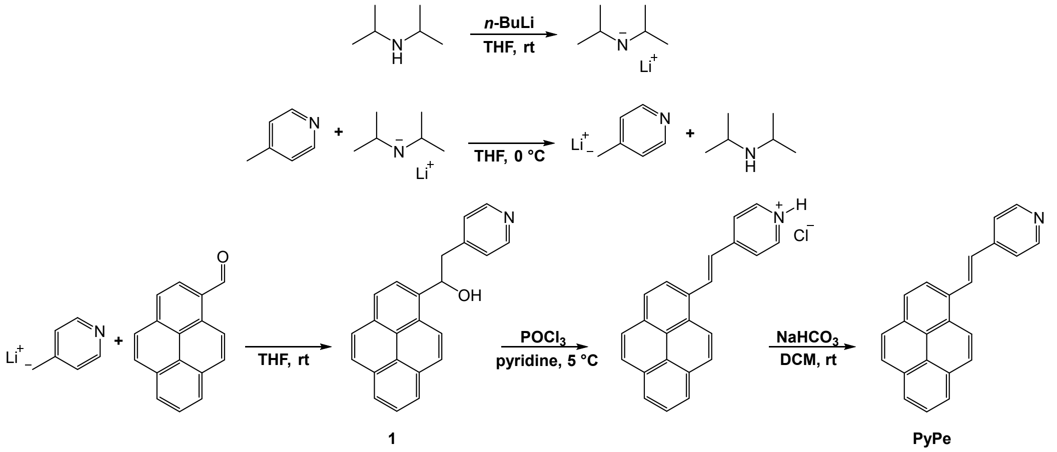

Figure 1. Synthetic route of PyPe.

2.1.2. Synthesis of the Complexes

Coordination compounds were obtained by mixing solutions of two equivalents of PyPe with zinc (II) iodide, cadmium (II) iodide, or mercury (II) iodide. Analysis through infrared spectroscopy (Figure S16) showed shifts in the transmittance bands between PyPe alone and the three complexes. The highest shifts (more than 10 cm^{-1}) occurred for bands associated to pyridine (in theory 1583, 1430, and 1030 cm^{-1}) or to pyrene (in theory 841 cm^{-1}) [28], consistent with binding between the pyridine moiety and metal ions (Figure 2).

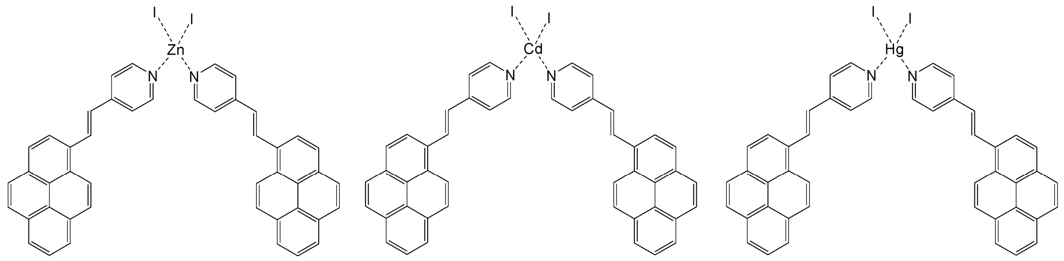

Figure 2. Proposed structures of complexes.

Single X-ray structures were obtained by layering a PyPe solution in $CHCl_3$ with a metal ions solution in THF. The three coordination compounds [M(PyPe)$_2$I$_2$], in which M = Zn (ZnPyPe), Cd (CdPyPe), and Hg (HgPyPe), respectively, were isostructural and crystallized in the monoclinic space group $P2_1/c$ with one complex per asymmetric unit (Figures 3a and S1–S12, Table S1). The metal ions had a distorted tetrahedral coordination, with τ_4 varying between 0.88 for the smallest cation, and 0.77 for mercury [29]. The tetrahedral geometry was characteristic of d^{10} metal ions; many complexes containing zinc, cadmium, or mercury ions adopt this configuration [30]. Moreover, the two ligands of a complex differed from each other according to the angle between the pyrene and pyridine planes, with ca. 10° for one ligand, and ca. 21.5° for the other ligand.

The M–I bonds were found to be slightly different with, on average, ca. 2.54 Å, 2.685 Å, and 2.64 Å for the ZnPyPe, CdPyPe, and HgPyPe complexes, respectively. The M–N distances within one compound were also slightly different and increased among the series, from ca. 2.07 Å for ZnPyPe, to ca. 2.29 Å for CdPyPe, and ca. 2.42 Å in HgPyPe. The bond lengths adopted an opposite trend compared to the angle between ligands and the central metal (ca. 91.8, 85.5, and 80.0°, respectively). These increasing bond lengths and decreasing angle trends among group 12 of metals have been observed in other works, such as by Chakraborti et al. [30], and are in accordance with the size of the metal ions; the smaller the ion is, the higher its charge density.

The complexes stack one on top of each other (Figure 3b), forming piles and allowing the pyridine moieties and the double bonds of one complex to form face-to-face π-π interactions of C–C = 3.3–3.4 Å with the pyrene of a neighbor complex (C7–C14, C30–C37). The stacks do not interact with each other, and form "zig-zag" assemblies (Figure 3d).

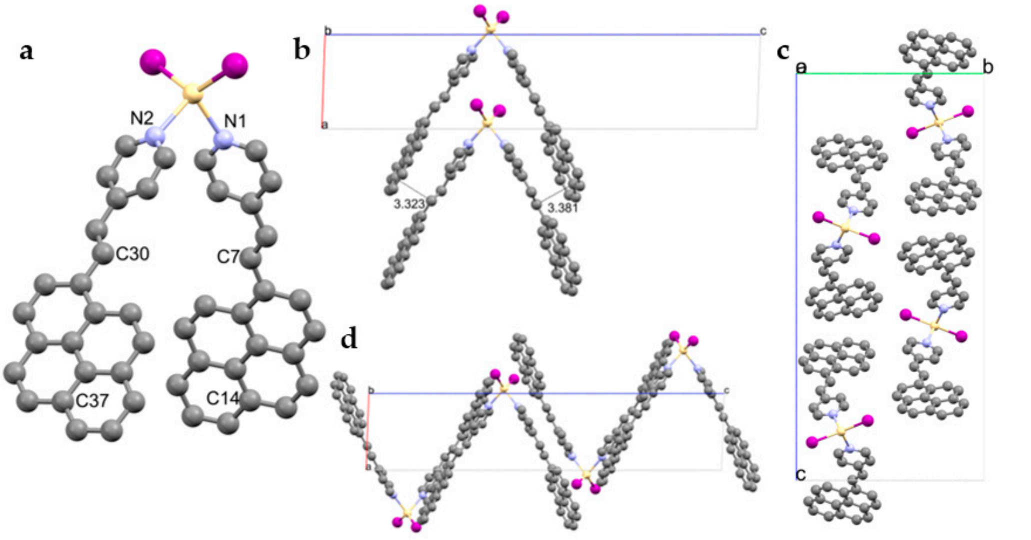

Figure 3. Solid state structure of CdPyPe as an example for MPyPe (all complexes being isostructural). (**a**) View of the complex with atoms of interest; (**b**) details along *b* axis with π-π stacking representation; (**c**) view of the asymmetric unit along *a* axis (H-atoms removed for clarity); (**d**) view of the asymmetric unit along *b* axis (H-atoms removed for clarity).

The detailed bond distances and angles for these three coordination compounds are given in Table 1; no other differences were observed between them.

Table 1. Main features of the three complexes, issued from their crystal structure.

Features [1]	ZnPyPe	CdPyPe	HgPyPe
Space group	$P2_1/c$	$P2_1/c$	$P2_1/c$
Coordination	Distorted tetrahedral (τ_4 = 0.88)	Distorted tetrahedral (τ_4 = 0.82)	Distorted tetrahedral (τ_4 = 0.77)
N1–M–N2	91.8°	85.5°	80.0°
M–N1	2.069(7) Å	2.289(10) Å	2.418(4) Å
M–N2	2.085(7) Å	2.300(10) Å	2.424(4) Å
M–I1	2.5384(11) Å	2.6804(12) Å	2.6343(4) Å
M–I2	2.5440(11) Å	2.6904(11) Å	2.6452 (4) Å
Torsion angle L1	11.7°	9.7°	9.5°
Torsion angle L2	23.6°	21.7°	20.5°
π-π interaction between two complexes	3.3–3.5 Å	3.3–3.4 Å	3.3–3.4 Å

[1] M = Zn, Cd, or Hg.

2.2. Compounds Stability

Because of the poor solubility of PyPe ligand in water, and to further design antimicrobial assays, the stability of the coordination compounds in water and bacterial culture medium with and without bacteria (*S. aureus*) was investigated (Figure 4). A precise mass of each compound was covered with 2.5 mL of water/medium, stirred, and the supernatant was sampled, filtered, and measured by ICP-OES over time to obtain the amount of metal ions (bound or released) present in the liquid phase. A first experiment was managed only in water, where the supernatant was entirely collected and analyzed through UV-visible spectrometry to determine the amount of PyPe that solubilized (both as free ligand and in coordinated forms; maximal absorption at 345 nm, Figure S14).

These UV-spectra showed no signal except a small absorption band (intensity lower than 0.15) for the non-coordinated ligand, indicating the poor solubility of our pyrene-pyridine derivatives. Therefore, due to the absence of ligand in the supernatant of coordination compounds (PyPe detection limit was approximated to be 0.4 µM), ICP-OES results (Figure 4) were interpreted as the amount of metal ions that were released from the complexes. It appeared that ZnPyPe was the least stable complex in these conditions, with about 13–18% of metal ions released after two weeks, while HgPyPe displayed almost no dissociation, and CdPyPe released about 2 to 5% cadmium iodide. These different dissociation rates showed similar trends to the solubility of the metal iodide salts themselves, which is 4.5 g/mL for zinc iodide [31], 0.8 g/mL for cadmium iodide [32], and only 38 pg/mL for mercury iodide [33].

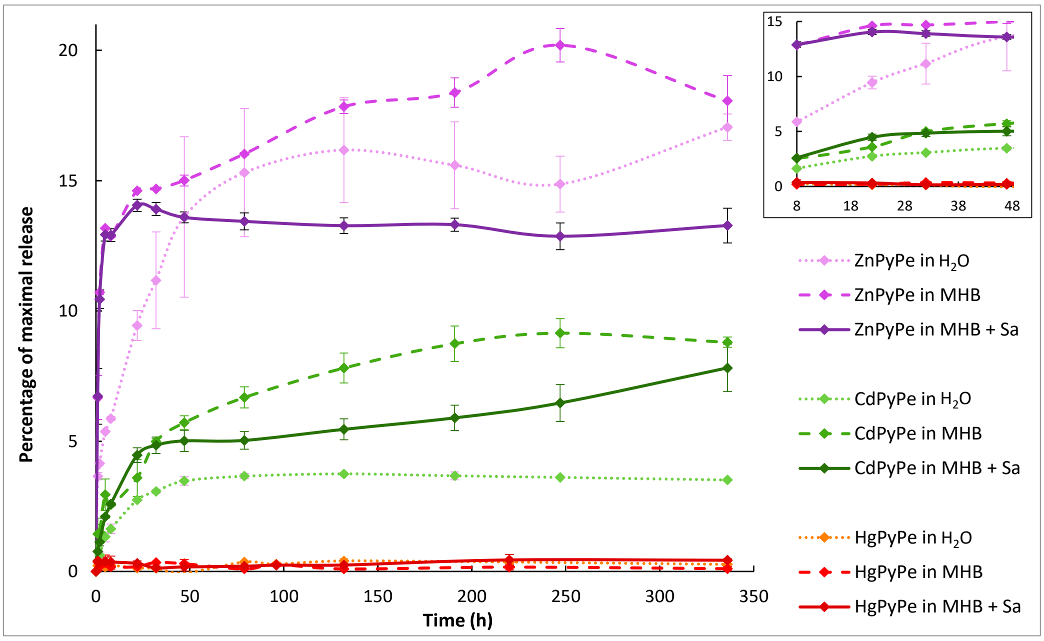

Figure 4. Plot of metal ion release in different media over time. The inserted plot zooms around 24 h of the experiment; MHB = Müller-Hinton Broth; Sa = *Staphylococcus aureus* 113 wildtype.

A notable difference was observed when the bacterial growth medium Müller-Hinton Broth (MHB) was used. Indeed, MHB is made of beef extract and contains several proteins able to chelate metal ions. The formation of these metal-protein complexes removed free metal ions from the medium and then promoted the further dissociation of coordination compounds.

Furthermore, when comparing the release in MHB with and without the presence of bacteria, the two curves initially overlapped and split after ca. 22 h. Then, in the presence of bacteria, the release reached a plateau (about 13% of zinc and 5% of cadmium ions released), whereas, in the sterile culture medium, the metal ion concentration continued to increase at a slower rate (up to 18% of zinc, and 8% of cadmium ions released). As the bacteria were removed from the supernatant during filtration, the difference is attributed to the amount of ions that penetrated into bacterial cells. This was hardly perceptible during the first hours of the experiment because of the exponential nature of the ion release and the low number of bacterial cells. The bacterial concentration usually reached a maximum after 20 to 24 h of incubation.

In contrast to zinc and cadmium, mercury (II) ions were not detectable in the supernatant (ICP-OES detection limit: 61 ppb), and we could not determine whether mercury was released and reprecipitated, or if it remained bound with the ligand.

2.3. *PyPe Solubility, Mixture and Complex Formation*

As PyPe and complexes were not so soluble in water, the next investigations focused on solubility in 9:1 H_2O/DMSO mixtures, intending to manage antibacterial assays under these conditions. DLS analysis of PyPe in different solvents (water, DMSO, THF, chloroform, and mixtures of these) demonstrated that it was not fully solubilized in DMSO. Instead, it formed nano-aggregates of about 55 nm diameter (Table S2), even though the solution looked homogeneous (transparent) and stable over weeks.

When the PyPe DMSO-dispersion was mixed with water, these aggregates were not dissolved but increased from about 55 to 80 nm in diameter. The coordination compounds formed even bigger aggregates in DMSO/water mixtures than PyPe (1.5 to 3-fold larger in diameter; see Table S2).

In DMSO/water mixtures, the size of the particles was globally unchanged following the addition of metal iodide salt solutions. This suggests that the metal ions are not able to diffuse inside these aggregates and to dissociate the aggregates, which requires placing two ligands in the right position to adopt the tetrahedral geometry observed on the crystal structures (see Figure 3 and Figures S1–12) that is characteristic of d^{10} metal ions. The complete coordination between metal ions and PyPe can thus not occur under these conditions, yet an adsorption of metal ions on the surface of these aggregates is not excluded. This would form some hydrophilic layer around the aggregates, stabilizing the interface with aqueous media.

On the other hand, THF and chloroform allowed full solubilization of PyPe (no detectable aggregates). Thus, interactions with metal ions can occur in this medium, explaining why coordination compounds and single crystals could be obtained from these solvents.

Finally, the metal iodide solutions with the PyPe nano-aggregates in water/DMSO mixtures enabled studying of the antibacterial behavior of the metal ions in presence of PyPe, but without the latter being coordinated. This is a key parameter for understanding the potential antibacterial effects of the coordination compounds by distinguishing between the action of the entire, non-dissociated coordination compound itself, and the released metal ions with free PyPe present.

2.4. Bioactivity of Metal-Organic Complexes

Antibacterial assays showed that PyPe alone is not an antibacterial compound at concentrations lower than 512 µM (Figure S17). Microdilution assays (for details, see part 4.7) against *S. aureus* 113 wildtype were then performed. A 96-well plate was prepared with solutions containing different concentrations of the complexes, their salts, or the 2:1 mixture PyPe/metal salt. After inoculation of the bacteria and 22 h of incubation, their growth was measured through their absorbance at 620 nm.

For the cadmium series (Figure 5 middle), the complexation or the presence of PyPe alone did not seem to have a strong influence, with a MIC equal to 4–8 µM (mixture) or 8–16 µM (salt and complex). Conclusions were more difficult to draw for zinc (Figure 5 left), as its status as an essential metal ion for some organisms, including mammals and bacteria, resulted in a much higher MIC than for other toxic metal ions (the literature gives values between 1 and 4 mM) [34,35]. At these concentrations, however, neither PyPe nor its zinc-complex were completely soluble, and the apparent MIC of ZnPyPe was associated with a too-high percentage of DMSO to be considered (12%; for DMSO survivability, see Figure S18). For the PyPe/ZnI_2 mixture, the MIC was a bit lower (250–500 µM), so the mixture seemed to be slightly more active than zinc iodide alone.

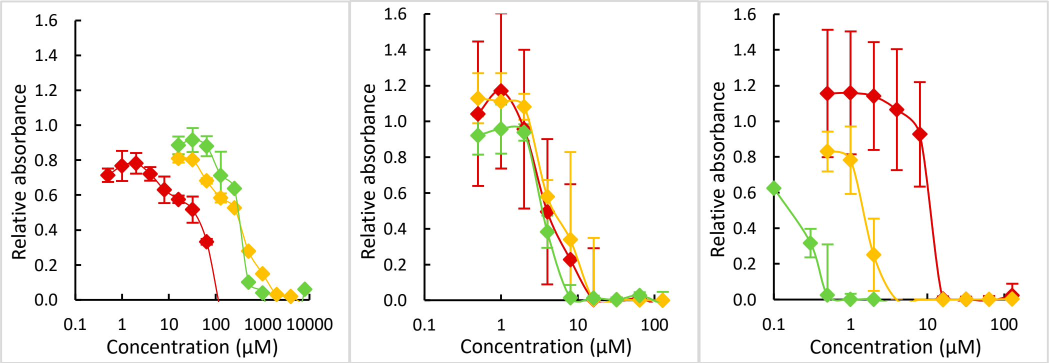

Figure 5. Plots of relative absorbance of bacteria, measured at 620 nm after 22 h of incubation, compared to the concentration of tested compounds. Data are the average of multiple experiments with n ≥ 9. Left: zinc series; middle: cadmium series; right: mercury series. The colors of the curves represent the growth of bacteria in the presence of complex (red), of the salt alone (orange), or the

mixture of metal iodide + 2 equivalents of PyPe (green). Negative values for the zinc series are due to poor solubility of ZnPyPe at high concentrations (>128 μM).

Finally, the results of the mercury series (Figure 5 right) are surprising: whereas the complex was less active than the salt alone, as can be expected given its poor solubility and release, the mixture PyPe/HgI_2 was 10-fold more active than mercury iodide, with a MIC between 0.2 and 0.5 μM versus 2 to 4 μM.

2.5. Bioactivity of Other Metal Ion-PyPe Mixtures

For other metal ion salts displaying antimicrobial properties, we could not obtain single crystals of their coordination compounds, but they were nevertheless tested in microdilution as salts and as mixtures with PyPe (Figure 6). The aim was to compare these effects to the apparent safety of PyPe alone. The studies were performed for silver (I) nitrate, gallium (III) nitrate, copper (II) nitrate, and bismuth (III) nitrate, with bismuth (III) subcitrate as a reference, as it has been an authorized drug since 2006 and is currently used to treat *Helicobacter pylori* infections [36] and has been tested as an adjuvant against resistant β-lactamases bacteria [5]. For all mixtures, the ratio of 1:2 metal salt to ligand was used.

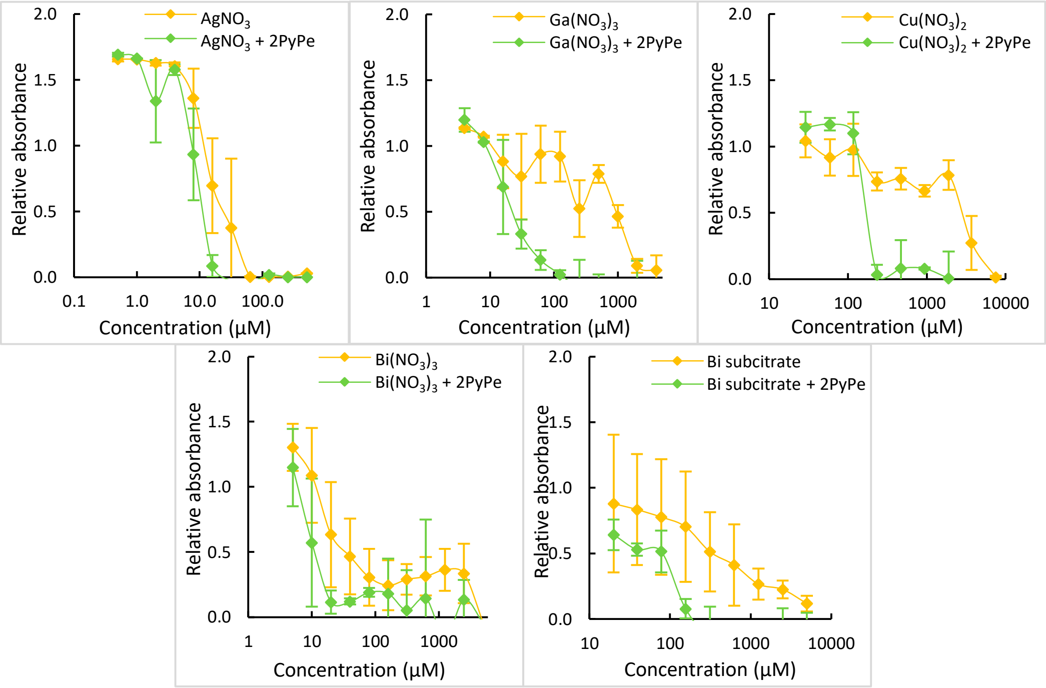

Figure 6. Plots of relative absorbance of bacteria, measured at 620 nm after 22 h of incubation, compared to the concentration of tested compounds. Data are the average of multiple experiments with n ≥ 9. Top: silver series (**left**); copper series (**middle**); gallium series (**right**). Bottom: bismuth nitrate series (**left**); bismuth subcitrate series (**right**).

Silver (I) nitrate assays against *S. aureus* 113wt resulted in a ca. four-fold decrease of the MIC in the presence of PyPe (between 8 to 16 μM instead of 32 to 64 μM) compared to the salt alone. While gallium (III) nitrate inhibited bacterial growth with a MIC between 1 to 2 mM, the addition of two equivalents of PyPe divided this concentration by a factor of 16, resulting in a MIC of 62–125 μM (and even between 31 and 62 μM for one of the triplicates). The same trend was observed for copper (II) nitrate, which alone displayed a MIC of more than 2.5 mM, whereas the presence of two equivalents of PyPe allowed a reduction by a factor of more than 20, reaching 120 to 240 μM.

For the assay with bismuth (III) nitrate, the MIC dropped from more than 2.5 mM for the salt alone to 10–20 μM in the presence of PyPe, reflecting a reduction by a factor of

more than 250, which was the best improvement observed in this work. For comparison, the MIC of bismuth (III) subcitrate alone was the same as the MIC of bismuth (III) nitrate, but the addition of two equivalents of PyPe to bismuth (III) subcitrate was less effective than for bismuth (III) nitrate, giving a division by only a factor of 30 (MIC ca. 80–160 µM).

3. Discussion

As PyPe is hardly soluble in water, a concentrated stock solution was prepared in DMSO, and mixed with bidistilled water up to appropriate working concentrations. These mixtures were analyzed through DLS, showing stable nano-dispersions, which surprisingly included the initial stock in DMSO. Comparing the solvents–water, DMSO, THF, and chloroform–it is noted that the aggregates of PyPe were smaller in DMSO than in DMSO/water mixtures (50 vs. 80 nm) and that no aggregates were found in THF or chloroform (see Table S2). The decrease in size followed the decrease in solvent polarity [37], suggesting that the formation of aggregates depends on the hydrophobic interactions between the aromatic moieties. Less polar solvents such as chloroform or THF were then able to interact with PyPe, breaking the hydrophobic interactions and dissolving PyPe completely, while the more polar solvent DMSO was less able to solubilize the aggregates. Water, due to being highly polar, was not able to interact with the aromatic groups and hence could not solubilize the pyrenes.

As they were hardly soluble in water, the coordination compounds released their metal ions over a couple of days. The release was better in Müller Hinton Broth culture medium compared to water, owing to the chelation effect of the contained proteins, which influenced the dissociation equilibrium of the compounds towards dissociation. In the presence of bacteria, both metal ions and complexes can, in principle, penetrate inside bacterial cells. As these bacteria were filtered off before the ICP measurement, the metal ions and complexes contained in the bacteria were excluded from the measurements. Therefore, the comparison of the values in sterile MHB and in MHB inoculated with *S. aureus* enabled us to estimate the proportion of metal ions that penetrated inside the bacteria. As explained in the *Results* section, ZnPyPe released over 14 days up to 18.1% of its zinc ions, of which 26.5% were assimilated by bacteria (18.1% detected in sterile medium; 13.3% detected in inoculated medium; 5% of total zinc amount was assimilated by bacteria, i.e., 26.5% of the released zinc). In comparison, 18.5% of released cadmium ions were assimilated by bacteria, clearly demonstrating that zinc had more facilities to penetrate inside bacterial cells than cadmium.

To explain this difference, one needs to consider the absorption pathways of metal ions in a bacterial cell: they mostly depend on the bacterial transporters and ion channels [38,39]. Indeed, ions are usually not able to cross through the bacterial cell membrane as their charge and polarity render them insoluble in the lipophilic membrane. Ion channels and transporters are, however, selective for those ions for which they are designed, and which are useful for bacteria [40]. Heavy and toxic metal ions such as cadmium(II) are typically not taken up easily, explaining their weak penetration rate. On the other hand, zinc(II) is an essential element for many, if not all, organisms. Therefore, zinc (II) ions can penetrate into the bacteria through usual pathways, such as zinc transporters or zinc channels [34,41], favoring their absorption. While the easy penetration of zinc ions appears to be advantageous, zinc is also better tolerated by bacteria, as shown by the antibacterial assays: the MIC of zinc iodide is much higher than the MIC for cadmium or mercury, thus raising solubility issues.

Comparing the results of the coordination compounds with the results of the metal salts shows that the complexation tends to increase MIC. The low release of metal ions seems then, unsurprisingly, to play a role in the high MIC of complexes, and is certainly associated with their low bioavailability due to the formation of nano-aggregates. Conversely, the results for the mixtures were better than the results of the metal salts alone, suggesting that the presence of PyPe tends to improve antibacterial activity, and hence

lower the MIC. Table 2 presents the MIC values of the different metal salts, their mixtures with ligand, and the complexes.

Table 2. MIC values of the compounds in μM (based on metal ion concentration), in increasing order from left to right.

**More Active Compounds**				**Less Active Compounds**
ZnI_2 + 2**PyPe**		ZnI_2		**ZnPyPe**
250–500	×4	1000–2000		solubility issue
CdI_2 + 2**PyPe**		CdI_2		**CdPyPe**
4–8	×2	8–16	=	8–16
HgI_2 + 2**PyPe**		HgI_2		**HgPyPe**
0.3–0.5	×10	2–4	×4	8–16
$AgNO_3$ + 2**PyPe**		$AgNO_3$		
8–16	×4	32–64		
$Ga(NO_3)_3$ + 2**PyPe**		$Ga(NO_3)_3$		
64–128	×16	1024–2048		
$Cu(NO_3)_2$ + 2**PyPe**		$Cu(NO_3)_2$		
120–240	>×20	MIC > 2500		
$Bi(NO_3)_3$ + 2**PyPe**		$Bi(NO_3)_3$		
10–20	>×250	MIC > 2500		
$Bi(subcitrate)_2$ + 2**PyPe**		$Bi(subcitrate)_2$		
80–160	>×30	MIC > 2500		

That all metal salts are less active against *S. aureus* alone than when they are co-administrated with PyPe supports the hypothesis that the presence of PyPe leads to a better penetration of the metal cations inside the bacterial cell. Indeed, the addition of lipophilic PyPe could temporarily form holes in the membrane and enable the entrance of metal ions into the bacteria. Such a formation of holes in cell membranes due to aromatic moieties is well documented for other molecules, e.g., pyrene, tryptophan, peptides, porphyrins, bipyridines, and substituted naphthols [38,42–45]. The hypothesis of PyPe forming holes in bacterial membranes could be confirmed by the improvement ranges relating to the MIC. Indeed, the highest improvement factors were related to those metal ions which are not supposed to, or not able to, enter into the bacteria through other mechanisms: zinc and to a lesser extent copper are essential metal ions, and bacteria express some transporters and/or channels to bring them inside (improvements: four- and 20-fold) [34,40,41]. Silver and copper ions are known to be able to penetrate through the bacterial membrane [46–48] (improvements: four- and 20-fold). On the other hand, bismuth, gallium, and mercury are not involved in bacterial metabolism and thus cannot easily enter bacterial cells by themselves. The formation of holes by PyPe nano-aggregates could, then, be the key step in their antibacterial action, as the presence of PyPe with these metal complexes improved MICs by factors of 10 to 250.

Finally, the lowest decrease in MIC for bismuth subcitrate with PyPe compared to bismuth (III) nitrate could be explained by the fact that bismuth (III) subcitrate is still an effective drug on its own, suggesting that the association between bismuth ions and subcitrate is still sufficient to successfully cross the bacterial cell membrane. Moreover, the affinity of Bi (III) for PyPe is perhaps weaker than its affinity for the subcitrate anion, and so charge compensation would be a strong factor here. The addition of PyPe did not have a key effect on bismuth subcitrate penetration, whereas in the case of bismuth (III) nitrate, its high solubility left free bismuth (III) cations, which were repelled from the lipophilic bacterial cell membrane.

4. Materials

1-pyrenecarboxaldehyde and 4-picoline were obtained from Fluorochem (Hadfield, UK). Phosphoryl chloride was purchased from Aldrich (Saint-Louis, MO, USA), di-isopropylamine from Acros Organics (Fisher Scientific Gmbh, Reinach, Switzerland), pyridine from Fisher Scientific Gmbh (Reinach, Switzerland), and all other chemicals were purchased from Sigma-Aldrich (Saint-Louis, MO, USA) and used as received if not otherwise stated.

^{1}H-NMR and ^{13}C-NMR spectra were recorded on a Avance III 400 MHz spectrometer (Bruker, Billerica, MA, USA) at room temperature with $CDCl_3$ or *d*$_6$-DMSO as solvents. Mass spectra were recorded on a Ion-Trap ESI-MS (Bruker, Billerica, MA, USA). Fluorescence spectra were measured on a Perkin Elmer instrument LS50B (Wellesley, MA, USA), and absorption spectra were collected on a Perkin Elmer UV/VIS Lambda 25 spectrometer (Wellesley, MA, USA). The ICP-OES was Perkin-Elmer Optima 7000DV equipment (Wellesley, MA, USA). Absorbance monitoring of bacterial growth was recorded on a plate reader Spark® multimode instrument from Tecan Trading AG (Männerdorf, Switzerland). Particle size distribution was determined by DLS using NanoLab 3D™ equipment from LS Instruments (Fribourg, Switzerland).

4.1. 1-(Pyren-1-yl)-2-(Pyridin-4-yl)Ethan-1-ol (1) Synthesis

10 mL of ice-cold dry THF was placed in a flask under argon, and 4-picoline (490 µL, 5 mmol, 1.0 eq) was added. Diisopropylamine (665 µL, 5 mmol, 1.0 eq), *n*-BuLi in hexane (3.3 mL, 1.6 M, 5 mmol, 1.0 eq), and dry THF (2 mL) were mixed into a dropping funnel, and the mixture was added dropwise at 0 °C over 25 min. The resulting mixture turned from yellowish to red. At the end of addition, the dropping funnel was rinsed with 2 mL of dry THF, and the dark red solution was stirred for 1 h at 0–5 °C (Figure 7).

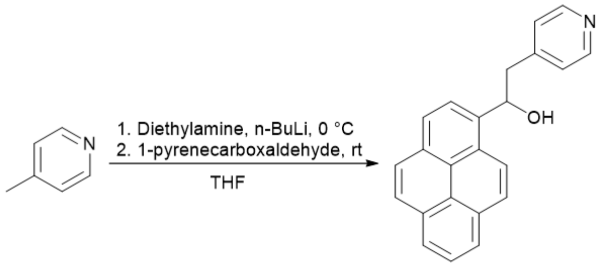

1

Figure 7. Synthesis step from 4-picoline to intermediate 1.

1-pyrenecarboxaldehyde (1.1 g, 5 mmol, 1.0 eq) was solubilized in 7 mL of dry THF under argon, and this green-brown solution was added dropwise to the previous one at 20 °C. Immediately after addition, a yellowish precipitate formed, and the solution turned brown. The resulting mixture was then stirred at room temperature overnight.

The orange solution was quenched with crushed ice, and after 10 min, THF was evaporated under reduced pressure. Then, 15 mL of distilled water was added to the yellow mixture, and the aqueous phase was extracted three times with about 20 mL of DCM. The combined organic layers were evaporated under reduced pressure, and a yellow oily solid was obtained, characterized by ^{1}H NMR (400 MHz, Chloroform-d) δ 8.50–8.45 (m, 2H), 8.31 (d, J = 9.3 Hz, 1H), 8.23–8.01 (m, 9H), 7.21–7.13 (m, 2H), and 6.03 (dd, J = 7.8, 5.0 Hz, 1H), 3.37–3.23 (m, 2H). ^{1}H NMR was not interpretable, but ESI-MS corresponded to the targeted product (calc. 323.4 *m*/*z*, found M+H^{+} 324.1 *m*/*z*), so the crude was used without further purifications.

4.2. (E)-4-(2-(Pyren-1-yl)Vinyl)Pyridine (PyPe) Synthesis

Compound **1** was sonicated in 10 mL of pyridine under argon to obtain a stable suspension. Then, phosphoryl chloride (700 µL, 7.5 mmol, 1.5 eq) in 6 mL pyridine was added

dropwise at 5 °C. After addition, the resulting red suspension was stirred at room temperature for 3 h. Then, the mixture was quenched with some pieces of crushed ice (vapors were formed) and stirred for 10 min. The solvent was evaporated under reduced pressure, forming a red sludge (Figure 8).

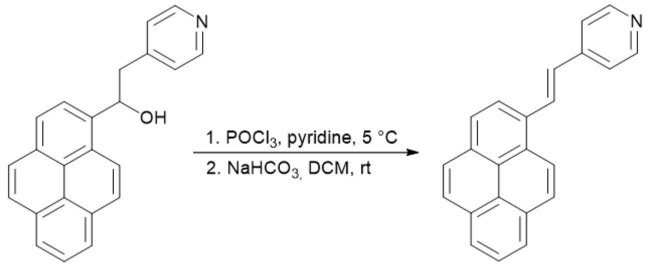

Figure 8. Synthesis step from intermediate 1 to PyPe.

About 30 mL each of DCM and acidified water (HCl, pH < 1) were added, and the organic layer was washed with acidified water. The non-dissolved solid was kept with the organic layer. The organic layer in DCM and red solid were vigorously shaken with 20 mL of sodium bicarbonate in water (1M), resulting in the formation of a beige precipitate. When all red color disappeared, the two layers were separated, and the aqueous layer was washed with DCM. The solvent was then evaporated under reduced pressure. From this, 1.0 g of beige solid (3.4 mmol, 68% total yield) was obtained, characterized by ^{1}H NMR (400 MHz, DMSO-d_6) δ 8.82 (d, J = 9.4 Hz, 1H, pyrene), 8.69 (d, J = 16.2 Hz, 1H, vinyl), 8.65–8.61 (m, 2H, pyridine), 8.56 (d, J = 8.2 Hz, 1H, pyrene), 8.36–8.32 (m, 3H, pyrene), 8.30 (d, J = 9.4 Hz, 1H, pyrene), 8.22 (m, 2H, pyrene), 8.10 (t, J = 7.6 Hz, 1H, pyrene), 7.87–7.82 (m, 2H, pyridine), 7.56 (d, J = 16.1 Hz, 1H, vinyl), and ESI-MS (calc. 305.1 *m*/*z*, found $M+H^+$ 306.2 *m*/*z*). ^{1}H NMR, IR, MS, and absorption and emission spectra are presented in Figures S16–S19.

4.3. Crystallization

ZnPyPe: A solution of PyPe at 10 mM in dry THF (30.5 mg, 100 µmol) was prepared, poured into a vial, and covered with 10 mL of a solution of zinc iodide at 5 mM in dry THF (16.0 mg, 50 µmol). The vials were sealed and left in the dark for three weeks. The supernatant was then removed, and the obtained orange needles (25.1 mg, 27 mmol, 54%) were washed with a small quantity of chloroform and analyzed via X-Ray diffraction. EA: (calculated) C, 59.41; H, 3.01, N, 3.25%; (found) C, 57.80; H, 2.93; N, 2.72.

A suitable crystal was selected and mounted on a loop with inert oil on a STADIVARI diffractometer. The crystal was kept at 250(2) K during data collection. Using Olex2 [49], the structure was solved with the SHELXT [50] structure solution program using Intrinsic Phasing and refined with the SHELXL [51] refinement package using Least Squares minimization. Crystal Data for $C_{46}H_{30}I_2N_2Zn$ (*M* =929.89 g/mol): monoclinic, space group $P2_1/n$ (no. 14), a = 7.1432(2) Å, b = 14.9839(3) Å, c = 33.7701(8) Å, β = 91.979(2)°, V = 3612.36(15) Å^3, Z = 4, T = 250 K, μ(Cu Kα) = 14.638 mm^{-1}, $Dcalc$ = 1.710 g/cm^3, 6410 reflections measured (5.24° ≤ 2Θ ≤ 136.31°), 6410 unique (R_{int} = /, R_{sigma} = 0.0647, twin 0.9694(14) : 0.0307(14)), which were used in all calculations. The final R_1 was 0.0690 (I > 2σ(I)) and wR_2 was 0.2103. CCDC-2290405.

CdPyPe: A solution of PyPe at 8 mM in chloroform/acetonitrile 1:5 (19.5 mg, 64 µmol) was prepared. Following this, 8 mL was poured into a vial and layered with 2.4 mL of a solution of cadmium iodide at 4 mM in methanol (23.4 mg, 32 µmol). The vials were sealed and left in the dark for three weeks. The supernatant was then removed, and the obtained orange needles (21.5 mg, 22.1 µmol, 69%) were washed with a small quantity of ethanol

and analyzed via X-Ray diffraction. EA: (calculated) C, 56.55; H, 3.10, N, 2.87% ; (found) C, 56.27; H, 2.73; N, 2.70.

A suitable crystal was selected and mounted on a loop with oil on a Stoe IPDS II diffractometer. The crystal was kept at 250(2) K during data collection. Using Olex2 [49], the structure was solved with the SHELXT [50] structure solution program using Intrinsic Phasing and refined with the SHELXL [51] refinement package using Least Squares minimization. Crystal Data for $C_{46}H_{30}CdI_2N_2$ (*M* =976.92 g/mol): monoclinic, space group $P2_1/n$ (no. 14), a = 7.1842(3) Å, b = 15.2855(5) Å, c = 33.4198(17) Å, β = 91.990(4)°, V = 3667.8(3) Å^3, Z = 4, T = 250(2) K, μ(MoKα) = 2.315 mm^{-1}, $Dcalc$ = 1.769 g/cm^3, 47945 reflections measured (2.44° ≤ 2Θ ≤ 53.7°), 7825 unique (R_{int} = 0.0740, R_{sigma} = 0.0390), which were used in all calculations. The final R_1 was 0.0391 (I > 2σ(I)), and wR_2 was 0.1089. CCDC-2290406.

HgPyPe: A solution of PyPe at 20 mM in chloroform (61.1 mg, 192 μmol) was prepared and 10 mL was poured into a vial and layered with 2.4 mL of a solution of mercury (II) iodide at 40 mM in dry THF (43.6 mg, 96 μmol). The vials were sealed and left in the dark for three weeks. The supernatant was then removed, and the obtained thin red needles (46.0 mg, 44 μmol, 45%) were washed with a small quantity of ethanol and analyzed via X-Ray diffraction. EA: (calculated) C, 51.87; H, 2.84, N, 2.63% ; (found) C, 51.90; H, 2.47; N, 2.42.

A suitable crystal was selected and mounted on a loop with oil on a Stoe IPDS II diffractometer. The crystal was kept at 250(2) K during data collection. Using Olex2 [49], the structure was solved with the SHELXT [50] structure solution program using Intrinsic Phasing and refined with the SHELXL [51] refinement package using Least Squares minimization. Crystal Data for $C_{46}H_{30}HgI_2N_2$ (*M* =1065.11 g/mol): monoclinic, space group $P2_1/n$ (no. 14), a = 7.1862(3) Å, b = 15.2968(4) Å, c = 33.2922(13) Å, β = 92.088(3)°, V = 3657.2(2) Å^3, Z = 4, T = 250(2) K, μ(MoKα) = 5.933 mm^{-1}, $Dcalc$ = 1.934 g/cm^3, 40578 reflections measured (2.45° ≤ 2Θ ≤ 52.44°), 7249 unique (R_{int} = 0.0720, R_{sigma} = 0.0398), which were used in all calculations. The final R_1 was 0.0298 (I > 2σ(I)), and wR_2 was 0.0635. CCDC-2290407.

Crystallographic data (CCDC number 2290405-2290407) have been deposited at the Cambridge Crystallographic Data Centre.

4.4. Stability of the Complexes

About 0.2–0.3 mg of metal complex (about 1 mg for HgPyPe, 2 mg for CdPyPe, and 5 mg for ZnPyPe) was precisely weighed and deposited in a well of a 24-well plate. This was done eight times for each complex, and the wells were then filled with 2.5 mL of distilled water (two times per complex), 2.5 mL of Müller Hinton Broth (two times per complex), and 2.5 mL of *S. aureus*-inoculated Müller Hinton Broth (four times per complex). The plate was then covered with tape and incubated in the plate reader, and the wells were regularly sampled (200 μL, replaced with fresh solvent). Samples were stored in the freezer until completion of the experiment, and then diluted with 5 mL of HNO_3 2% in water and filtered over 0.45 μm filters. The resulting metal concentration was measured with ICP.

About 0.2–0.3 mg of metal complex form (about 1 mg for HgPyPe, 2 mg for CdPyPe, 5 mg for ZnPyPe, or 7 mg of PyPe) was precisely weighed and deposited in a 5 mL vial. This was done three times for each compound, and the vials were then filled with 2.5 mL of distilled water. The vials were then sealed and shaken at 37 °C, 180 rpm. The supernatant was sampled after 2, 7, and 24 h and analyzed via UV-visible spectrometry from 200 to 800 nm.

4.5. Dynamic Light Scattering

A concentrated solution of PyPe 20 mM in DMSO, and dilutions to 1 mM, 100 μM, 10 μM, and 1 μM in bidistilled water were analyzed in DLS, 10 repetitions.

4.6. Microdilution Assays

Stock solutions of the tested compounds in Müller Hinton Broth (metal salts) or DMSO (PyPe and complexes) were prepared: complexes were stored at 830 μM (ZnPyPe), 570 μM (CdPyPe), and 490 μM (HgPyPe) in DMSO (maximal solubility); stock dispersion of PyPe was at 40 mM in DMSO/H_2O 7:3.

These solutions were diluted using MHB to the working concentration. Attention was paid to always having the same quantity of DMSO in all paired solutions (metal salt solution alone and with two equivalents of PyPe). This was achieved by always adding into the metal salt solution the precise amount of DMSO that the mixture PyPe/metal salt would have contained at this concentration. For instance, concerning silver tested from 0.5 to 512 μM ($AgNO_3$ + 2PyPe), the working concentration of the mixture was 1024 μM for silver nitrate and 2048 μM for PyPe. Two milliliters of the mixture contained, then, 102.4 μL of PyPe stock dispersion at 70% of DMSO, i.e., 3.6% of DMSO. When the stock solution of silver nitrate was diluted to test the metal salt alone, 3.6% of DMSO was added to the non-diluted solution for comparative results. Then, a range of different dilutions was prepared. The final ratio of DMSO is described in Table S3. Equal volumes of these dilutions were tested in quadruplicates in a 96-well plate. Three wells of these quadruplicates were then inoculated with equal volumes of a bacteria culture around 2.10^5 CFU/mL, and all fourth wells were used as sterility controls and for background measurement, in which the bacteria culture was replaced by sterile MHB. The bacterial growth was then followed through absorbance monitoring at 620 nm in a plate reader, in which the 96-well plate was shaken and kept at 37 °C for 23 h. Relative absorbance was calculated using the difference in absorbance between the concerned well and the background (MH broth with same metal ion and DMSO concentrations). Wells where the DMSO amount was higher than 5% were not taken into account for MIC, as bacterial death in these wells could have been due to DMSO (see Figure S18, DMSO survivability).

5. Conclusions

This work describes the synthesis of a pyrene-pyridine ligand and its three tetrahedral complexes based on zinc, cadmium, and mercury iodide. Due to poor solubility, these four compounds form nano-aggregates in DMSO and water/DMSO mixtures (about 80 nm of diameter for PyPe alone), likely due to strong binding of both the metal ions by the pyridine moiety and the pyrenes through π-π interactions. This leads to reduced bioavailability of the metal ions, with only 13% of zinc and 5% of cadmium being released over 22 h, and consequently, the MIC of these compounds is higher than the MIC of the metal salt alone (multiplied by four for HgPyPe).

However, the antimicrobial experiments show that the addition of two equivalents of PyPe to metal ion solutions, not leading to any complexation, results in a decrease in the MIC, ranging from a factor of two (zinc and cadmium iodide) through to 250 (bismuth nitrate). These mixtures show, then, a strong improvement in the bioactivity of the metal salts. An interesting perspective would be to find good conditions for the synthesis of complexes between PyPe and other tested metal ions, such as silver (I), bismuth (III), copper (II), or gallium (III), but all assays carried out so far have failed. Their mixture with two equivalents of PyPe is however already more active than the metal salt alone, and it is expected that their complexes would follow the same trend as the mercury and cadmium complexes (retain metal ions and reduce bioavailability). One can then imagine combining mixtures of metal salt/PyPe for short-term antibacterial effects, and with PyPe complexes for longer-term effects through release.

Because they currently encounter less bacterial resistance, metal ions are of renewed interest to the scientific community. To avoid the emergence at large scale of new antimicrobial resistances, they should be used in the smallest possible quantities. Their use as adjuvants in mixtures or complexes results in antibacterial synergy and is therefore a good path in the right direction. On the other hand, whereas medicine requires new kinds of antibiotics to treat multiresistant infections, it is unfortunate that some of these metal ions are avoided because of a too-small therapeutic window. These antibacterial experiments

clearly show that an association between PyPe and metal salts facilitates the antimicrobial effects of metal ions. In the future, the possibility of modulating the antimicrobial or even toxic properties of metal ions could become a great tool in this period of expansion of antibiotic resistance.

Supplementary Materials: The following supporting information can be downloaded at: https://www.mdpi.com/article/10.3390/land13040442/s1, Figures S1–S3, S5–S7 and S9–S11: Solid state structures of ZnPyPe, CdPyPe, and HgPyPe; Figures S4, S8 and S12: P-XRD spectra of ZnPyPe, CdPyPe, and HgPyPe; Table S1: Crystal data and structure refinement of CdPyPe, HgPyPe and ZnPyPe, Figure S13: Infrared spectra of PyPe, ZnPyPe, CdPyPe, and HgPyPe; Figure S14: absorption and emission spectra of PyPe; Figure S15: ^{1}H NMR spectra of PyPe; Figure S16: ^{1}H NMR spectra of PyPe coordination compounds; Figure S17: ESI-MS spectra of PyPe; Table S2: Hydrodynamic diameter of PyPe aggregates, observed by DLS; Figure S18: Plot of relative absorbance of *S.aureus* 113wt in presence of PyPe and DMSO; Table S3: DMSO percentage in diluted solutions for antibacterial tests; Figure S19: solubility of PyPe derivatives.

Author Contributions: Conceptualization, funding acquisition, project administration, supervision and writing—review and editing, K.M.F. SCXRD analysis, A.C. Data curation, formal analysis, investigation, methodology, validation, visualization, writing—original draft, J.V.S. All authors have read and agreed to the published version of the manuscript.

Funding: This research was funded by the Swiss National Science Foundation (SNF, grant n° 178827), FriMat, the NCCR Bioinspired Material, and the University of Fribourg.

Data Availability Statement: Crystallographic data (CCDC number 2290405-2290407) have been deposited at the Cambridge Crystallographic Data Centre.

Acknowledgments: The authors thank Bernd Giese for fruitful discussions.

Conflicts of Interest: The authors declare no conflicts of interest.

References

1. Martin, M.J.; Thottathil, S.E.; Newman, T.B. Antibiotics Overuse in Animal Agriculture: A Call to Action for Health Care Providers. *Am. J. Public Health* **2015**, *105*, 2409–2410.
2. Dixit, A.; Kumar, N.; Kumar, S.; Trigun, V. Antimicrobial Resistance: Progress in the Decade since Emergence of New Delhi Metallo-β-Lactamase in India. *Indian J. Community Med.* **2019**, *44*, 4.
3. Butler, M.S.; Blaskovich, M.A.T.; Cooper, M.A. Antibiotics in the Pipeline at the End of 2015. *J. Antibio.* **2017**, *70*, 3–24.
4. Sun, H. *Biological Chemistry of Arsenic, Antimony and Bismuth*; John Wiley & Sons, Ltd.: Chichester, UK, 2011.
5. Wang, R.; Lai, T.P.; Gao, P.; Zhang, H.; Ho, P.L.; Woo, P.C.Y.; Ma, G.; Kao, R.Y.T.; Li, H.; Sun, H. Bismuth Antimicrobial Drugs Serve as Broad-Spectrum Metallo-β-Lactamase Inhibitors. *Nat. Commun.* **2018**, *9*, 439.
6. Shareena Dasari, T.P.; Zhang, Y.; Yu, H. Antibacterial Activity and Cytotoxicity of Gold (I) and (III) Ions and Gold Nanoparticles. *Biochem. Pharmacol.* **2015**, *4*, 199.
7. Abram, S.L.; Gagnon, J.; Priebe, M.; Hérault, N.; Fromm, K.M. Ag Nanoencapsulation for Antimicrobial Applications. *Chimia* **2018**, *72*, 249–252.
8. Fromm, K.M. Give Silver a Shine. *Nat. Chem.* **2011**, *3*, 178.
9. Ben Miloud, S.; Ali, M.M.; Boutiba, I.; Van Houdt, R.; Chouchani, C. First Report of Cross Resistance to Silver and Antibiotics in Klebsiella Pneumoniae Isolated from Patients and Polluted Water in Tunisia. *Water Environ. J.* **2021**, *35*, 730–739.
10. Gómez-Sagasti, M.T.; Becerril, J.M.; Epelde, L.; Alkorta, I.; Garbisu, C. Early Gene Expression in Pseudomonas Fluorescens Exposed to a Polymetallic Solution. *Cell Biol. Toxicol.* **2015**, *31*, 39–81.
11. Delmar, J.A.; Su, C.C.; Yu, E.W. Bacterial Multidrug Efflux Transporters. *Annu. Rev. Biophys.* **2014**, 43, 93–117.
12. Hobman, J.L.; Crossman, L.C. Bacterial Antimicrobial Metal Ion Resistance. *J. Med. Microbiol.* **2015**, *64*, 471–497.
13. Bernhoft, R.A. Mercury Toxicity and Treatment: A Review of the Literature. *J. Environ. Public Health* **2012**, *2012*, 460508.
14. Carver, P.L. *Chapter 1 in Essential Metals in Medicine: Therapeutic Use and Toxicity of Metal Ions in Clinic*; De Gruyter, C.P., Ed.; Walter de Gruyter GmbH & Co. KG: Berlin, Germany, 2019.
15. Crisponi, G.; Nurchi, V.M. Metal Ion Toxicity. In *Encyclopedia of Inorganic and Bioinorganic Chemistry*; John Wiley & Sons, Ltd.: Hoboken, NJ, USA, 2015; pp. 1–14.
16. Nurchi, V.M.; Djordjevic, A.B.; Crisponi, G.; Alexander, J.; Bjorklund, G.; Aaseth, J. Arsenic toxicity: Molecular Targets and Therapeutic Agents. *Biomolecules* **2020**, *10*, 235.
17. Jones, M.M.; Vaughn, W.K. HSAB Theory and Acute Metal Ion Toxicity and Detoxification Processes. *J. Inorg. Nucl. Chem.* **1978**, *40*, 2081–2088.

18. Pahan, K.; Gachhui, R.; Ray, S.; Chaudhuri, J.; Mandal, A. Bacterial Degradation and Utilization of Merbromine and Fluorescein Mercuric Acetate. *Bull. Environ. Contam. Toxicol.* **1992**, *48*, 421–427.
19. Russell, A.D. The Role of Plasmids in Bacterial Resistance to Antiseptics, Disinfectants and Preservatives. *J. Hosp. Infect.* **1985**, *6*, 9–19.
20. Clements, C.J.; Ball, L.K.; Ball, R.; Pratt, D. Thiomersal in Vaccines. *Lancet* **2000**, *355*, 1279–1280.
21. Nogueira, L.; Tracey, A.T.; Alvim, R.; Reisz, P.; Scherz, A.; Coleman, J.A.; Kim, K. Developments in Vascular-Targeted Photodynamic Therapy for Urologic Malignancies. *Molecules* **2020**, *25*, 5417.
22. Walker, J.; Saravia, N.G. Inhibition of Leishmania Donovani Promastigote DNA Topoisomerase I and Human Monocyte DNA Topoisomerases I and II by Antimonial Drugs and Classical Antitopoisomerase Agents. *J. Parasitol.* **2004**, *90*, 1155–1162.
23. Nadar, V.S.; Chen, J.; Dheeman, D.S.; Galván, A.E.; Yoshinaga-Sakurai, K.; Kandavelu, P.; Sankaran, B.; Kuramata, M.; Ishikawa, S.; Rosen, B.P.; et al. Arsinothricin, an arsenic-containing non-proteinogenic amino acid analog of glutamate, is a broad-spectrum antibiotic. *Commun Biol.* **2019**, *2*, 131.
24. Frei, A.; Verderosa, A.D.; Elliott, A.G.; Zuegg, J.; Blaskovich, M.A.T. Metals to Combat Antimicrobial Resistance. *Nat. Rev. Chem.* **2023**, *7*, 202–204.
25. Gasser, G.; Metzler-Nolte, N. The Potential of Organometallic Complexes in Medicinal Chemistry. *Curr. Opin. Chem. Biol.* **2012**, *16*, 84–91.
26. Claudel, M.; Schwarte, J.V.; Fromm, K.M. New Antimicrobial Strategies Based on Metal Complexes. *Chemistry* **2020**, *2*, 849–899.
27. Sakaran, N.; Das, A.; Samanta, A. Interaction between a pyridyl and a naphthyl/pyrenyl moiety in covalently linked systems. *Chem. Phys. Lett.* **2002**, *351*, 61–70.
28. National Institute of Standards and Technologies. *IR Spectra of Pyridine and Pyrene*. Available online: https://webbook.nist.gov/cgi/cbook.cgi?ID=C129000&Type=IR-SPEC&Index=1#IR-SPEC and https://webbook.nist.gov/cgi/cbook.cgi?ID=C110861&Units=SI&Type=IR-SPEC&Index=3#IR-SPEC (accessed on 10 September 2023)
29. Yang, L.; Powell, D.R.; Houser, R.P. Structural Variation in Copper (I) Complexes with Pyridylmethylamide Ligands: Structural Analysis with a New Four-Coordinate Geometry Index, τ_4. *Dalt. Trans.* **2007**, *9*, 955–964.
30. Chakraborty, S.; Purkayastha, D.D.; Das, G.; Bhattacharjee, C.R.; Mondal, P.; Prasad, S.K.; Shankar Rao, D.S. *Polyhedron* **2016**, *105*, 150–158.
31. Fisher Scientific, Zinc (II) Iodide Pure 98+%. Available online: https://www.fishersci.ch/shop/products/zinc-iodide-98-pure-thermo-scientific/10686782#?keyword=zinc%20iodide (accessed on 10 September 2023).
32. Fisher Scientific, Cadmium (II) Iodide Acros Organics 99%. Available online: https://www.fishersci.ch/shop/products/cadmium-iodide-99-acros-organics/10402855/en&usg=AOvVaw0xD1K2FeabOfppJDdB7tHJ&opi=89978449 (accessed on 17 December 2023).
33. Lide, D.R. (Ed.) Section 8 Analytical Chemistry. In *Handbook of Chemistry and Physics*, 77th ed.; CRC Press: Boca Raton, FL, USA, 1996; p. 8.92.
34. Hantke, K. Bacterial Zinc Uptake and Regulators. *Curr. Opin. Microbiol.* **2005**, *8*, 196–202.
35. Pormohammad, A.; Turner, R.J. Silver Antibacterial Synergism Activities with Eight Other Metal(Loid)-Based Antimicrobials against *Escherichia coli*, *Pseudomonas aeruginosa*, and *Staphylococcus aureus*. *Antibiotics* **2020**, *9*, 853.
36. Lambert, J.R.; Midolo, P. The Actions of Bismuth in the Treatment of Helicobacter Pylori Infection. *Aliment. Pharmacol. Ther.* **1997**, *11*, 27–33.
37. Fisher Scientific, Summary of Key Physical Data for Solvents. Available online: https://www.fishersci.co.uk/gb/en/scientific-products/technical-tools/summary-key-physical-data-solvents.html (accessed on 7 January 2024).
38. Yang, K.; Kotak, H.A.; Haynes, C.J.E. Metal-Organic Ion Transport Systems. *Coord. Chem. Rev.* **2022**, *470*, 214705.
39. Booth, I.R.; Edwards, M.D.; Miller, S. Bacterial Ion Channels. *Biochemistry* **2003**, *42*, 10045–10053.
40. Fritsch, S.; Ivanov, I.; Wang, H.; Cheng, X. Ion Selectivity Mechanism in a Bacterial Pentameric Ligand-Gated Ion Channel. *Biophys. J.* **2011**, *100*, 390–398.
41. Hantke, K. Bacterial Zinc Transporters and Regulators. In *Zinc Biochemistry, Physiology, and Homeostasis: Recent Insights and Current Trends*; Springer: Dordrecht, Germany, 2001; pp. 53–63.
42. Vermaas, J.V.; Crowley, M.F.; Beckham, G.T. Molecular Simulation of Lignin-Related Aromatic Compound Permeation through Gram-Negative Bacterial Outer Membranes. *J. Biol. Chem.* **2022**, *298*, 102627.
43. Zhang, J.; Liu, S.; Li, H.; Tian, X.; Li, X. Tryptophan-Based Self-Assembling Peptides with Bacterial Flocculation and Antimicrobial Properties. *Langmuir* **2020**, *36*, 11316–11323.
44. Espeche, J.C.; Varas, R.; Maturana, P.; Cutro, A.C.; Maffía, P.C.; Hollmann, A. Membrane Permeability and Antimicrobial Peptides: Much More than Just Making a Hole. *Pept. Sci.* **2023**, *116*, e24305.
45. Gao, H.; Wu, M.; Liu, H.; Zhang, T.; Zhang, X. Cell Toxic Damages during Polycyclic Aromatic Hydrocarbons Biodegradation by Pseudomonas Aeruginosa G24. *J. Water Process Eng.* **2023**, *54*, 103992.
46. Santo, C.E.; Lam, E.W.; Elowsky, C.G.; Quaranta, D.; Domaille, D.W.; Chang, C.J.; Grass, G. Bacterial killing by dry metallic copper surfaces. *Appl. Env. Microbio.* **2011**, *77*, 794–802.
47. Thurman, R.; Gerba, C.P. The molecular mechanisms of copper and silver ions disinfection of bacteria and viruses. *Crit. Rev. Env. Sci. Technol.* **1989**, *18*, 295–315.
48. Silvestry-Rodriguez, N.; Sicairos-Ruelas, E.E.; Gerba, C.P.; Bright, K.R. Silver as Disinfectant. *Rev. Env. Cont. Toxicol.* **2007**, *191*, 23.

49. Dolomanov, O.V.; Bourhis, L.J.; Gildea, R.J.; Howard, J.A.K.; Puschmann, H.J. OLEX2: A Complete Structure Solution, Refinement and Analysis Program. *Appl. Cryst.* **2009**, *42*, 339–341.
50. Sheldrick, G.M. SHELXT-Integrated Space-Group and Crystal-Structure Determination. *Acta Cryst.* **2015**, *A71*, 3–8.
51. Sheldrick, G.M. Crystal Structure Refinement with SHELXL. *Acta Cryst.* **2015**, C71, 3–8.

Disclaimer/Publisher's Note: The statements, opinions and data contained in all publications are solely those of the individual author(s) and contributor(s) and not of MDPI and/or the editor(s). MDPI and/or the editor(s) disclaim responsibility for any injury to people or property resulting from any ideas, methods, instructions or products referred to in the content.
